# Glycosylated superparamagnetic nanoparticle gradients for osteochondral tissue engineering

**DOI:** 10.1016/j.biomaterials.2018.05.029

**Published:** 2018-09

**Authors:** Chunching Li, James PK. Armstrong, Isaac J. Pence, Worrapong Kit-Anan, Jennifer L. Puetzer, Sara Correia Carreira, Axel C. Moore, Molly M. Stevens

**Affiliations:** a Department of Materials, Department of Bioengineering and Institute of Biomedical Engineering, Imperial College London, Prince Consort Road, London, SW7 2AZ, United Kingdom; b H. H. Wills Physics Laboratory, University of Bristol, Tyndall Avenue, Bristol, BS8 1TL, United Kingdom

**Keywords:** Magnetic, Nanoparticles, Gradients, Osteochondral, Tissue engineering

## Abstract

In developmental biology, gradients of bioactive signals direct the formation of structural transitions in tissue that are key to physiological function. Failure to reproduce these native features in an *in vitro* setting can severely limit the success of bioengineered tissue constructs. In this report, we introduce a facile and rapid platform that uses magnetic field alignment of glycosylated superparamagnetic iron oxide nanoparticles, pre-loaded with growth factors, to pattern biochemical gradients into a range of biomaterial systems. Gradients of bone morphogenetic protein 2 in agarose hydrogels were used to spatially direct the osteogenesis of human mesenchymal stem cells and generate robust osteochondral tissue constructs exhibiting a clear mineral transition from bone to cartilage. Interestingly, the smooth gradients in growth factor concentration gave rise to biologically-relevant, emergent structural features, including a tidemark transition demarcating mineralized and non-mineralized tissue and an osteochondral interface rich in hypertrophic chondrocytes. This platform technology offers great versatility and provides an exciting new opportunity for overcoming a range of interfacial tissue engineering challenges.

## Graphical abstract

**Figure:**
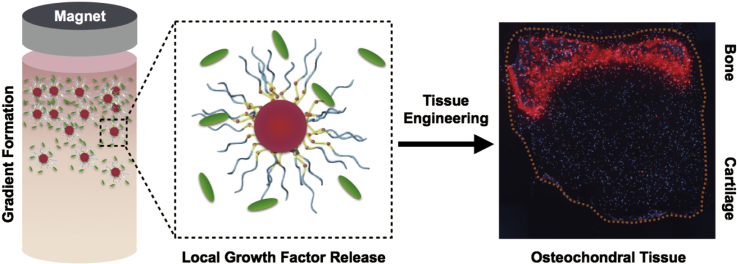

## 1 Introduction

Biological gradients play an essential role in defining the functional role of a wide range of tissues, including tendon, cartilage and the central nervous system [[1], [2], [3]]. These gradients are largely dictated by an anisotropic distribution of growth factors present during embryological development and pre-adolescent growth. For instance, the formation of the cartilage-bone interface in osteochondral tissue is heavily influenced by a polarized distribution of pro-osteogenic growth factors, such as bone morphogenetic protein 2 (BMP-2) [3,4]. These local differences in growth factor concentration produce spatial variance in cell signaling, which can, in turn, lead to distinct cell phenotypes across the tissue (*e.g.* osteoblasts and chondrocytes). In a structural system such as osteochondral tissue, the different cell types sculpt the final tissue composition *via* the production and remodeling of different extracellular matrix components. These structural features play a critically important role in the functional performance of the tissue; for instance, the gradual transition between bone and cartilage enables the smooth transmission and distribution of compressive loads through the osteochondral tissue [5,6].

Despite these well-known considerations, the overwhelming majority of *in vitro* engineering strategies use uniform scaffolds and homogeneous growth factor delivery to produce isotropic tissue constructs. It is clear that more sophisticated fabrication processes are required to replicate the native complexity and fulfill the functional requirements of tissue grafts. A few material strategies have been developed that can heterogeneously deliver biological or mechanical cues [[7], [8], [9], [10], [11]]. A simplistic strategy, which has found clinical application, is to laminate separate biomaterials by suture or other approaches such as using a small amount of solvent after preparation [4,12]. This approach uses the material composition and structure of the different scaffold layers to direct cell fate and tissue formation, however, discontinuities at the interface make these biphasic materials highly susceptible to delamination [8]. A smoother transition can be achieved using a gradient maker, in which microspheres laden with growth factors are distributed in graded fashion throughout a hydrogel [13], or through specialist techniques, such as photo-patterning [14] and microfluidics [14]. These systems can produce excellent gradients, but can often be limited by complex micro-fabrication procedures or compatibility with different material systems. It is clear that there is an urgent and unmet need for a simple and versatile gradient casting method that can be universally applied to different biomaterial and tissue engineering protocols.

**Fig. 1:**
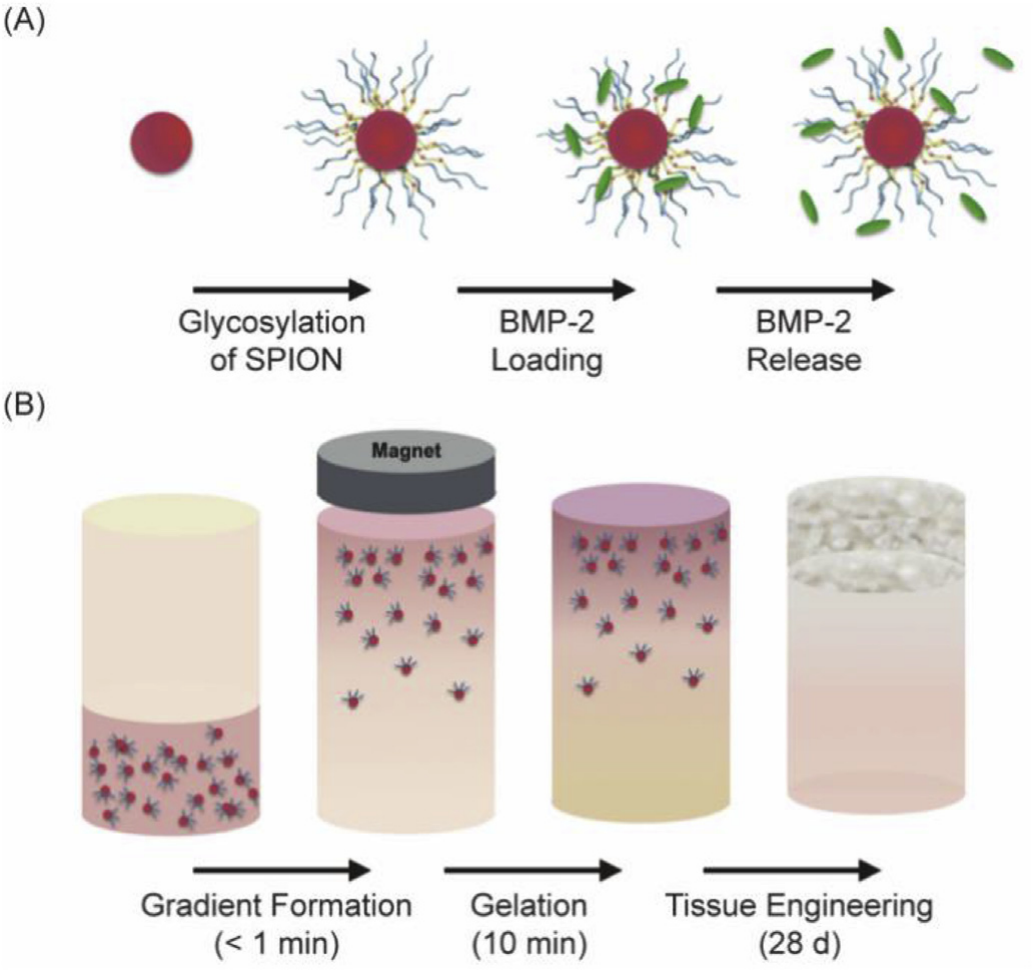
Engineering osteochondral tissue using magnetically-aligned glycosylated SPIONs. (A) SPIONs are conjugated with heparin to produce a glycosylated corona that can efficiently sequester and release growth factors. (B) An external magnetic field is used to field-align glycosylated SPIONs in a hMSC-laden agarose hydrogel, which is thermally gelled and cultured for 28 days to generate robust osteochondral constructs comprising both bone and cartilage tissue.

To this end, we present a new strategy in which externally-applied magnetic fields are used to decorate different biomaterials with continuous gradients of growth factor loaded glycosylated superparamagnetic iron oxide nanoparticles (SPIONs) (Fig. 1). SPIONs have previously been used to generate polarization of the signaling protein RanGTP within *Xenopus* egg extracts to spatially modify the assembly of microtubule networks [15]. To the best of our knowledge, however, SPIONs have not yet been investigated as a method for patterning biochemical gradients for *in vitro* tissue engineering. Here, we report the use of magnetically-aligned gradients of BMP-2 to spatially direct mineralization during osteochondral tissue engineering. Specifically, we used an external magnetic field to pattern glycosylated SPIONs into an agarose hydrogel, pre-laden with human mesenchymal stem cells (hMSCs). Thermal gelation of the hydrogel enabled us to stably encapsulate a BMP-2 gradient, which was used to spatially stimulate osteogenic gene expression and tissue mineralization over a 28-day culture. The resulting tissues exhibited a cartilage region, rich in type II collagen and glycosaminoglycan, with a transition into a mineralized, bone region exhibiting extensive distribution of β-tricalcium phosphate (β-TCP) and hydroxyapatite (HAP). In certain cases, these differences in extracellular matrix provided an increased compressive instantaneous modulus at the bone region compared to the cartilage region. This approach required no specialized equipment, other than a magnet, and offered great versatility in patterning different hydrogel or scaffold systems. The ability to produce tissue-scale biochemical gradients in under a minute across different biomaterials should afford this platform technology wide applicability across a range of complex tissue engineering systems.

## 2 Materials and methods

### 2.1 Glycosylated SPION synthesis and characterization

A 0.9 mL solution of 1.5 mg mL^−1^ of heparin (Sigma Aldrich, UK) was prepared in pH 5.4 4-morpholineethanesulfonic acid buffer (MES, Thermo Fisher, USA), and mixed for 15 min with 5 mg of *N*-(3-dimethylaminopropyl)-*N*′-ethylcarbodiimide hydrochloride (EDC, Sigma Aldrich, UK) and 1 mg of *N*-hydroxysuccinimide (NHS, Sigma Aldrich, UK). 0.1 mL of 5 mg mL^−1^ amine-functionalized 20 nm SPIONs (SHA-20, Ocean NanoTech, USA) were then added and mixed for 24 h at room temperature. After the conjugation, the product was dialyzed against 5 L of phosphate-buffered saline (PBS, Thermo Fisher, USA) for 3 days with two daily buffer changes to ensure complete removal of unconjugated heparin. The conjugated SPIONs were then concentrated to 2 mg mL^−1^ using 100 kDa cut-off Amicon ultra-centrifugal filter unit (Merck Millipore, USA).

Zeta potential and hydrodynamic diameter measurements were performed using a Zetasizer Zen 3600 (Malvern Instruments, UK), with samples in pH 7 deionized water at a concentration of 40 μg mL^−1^. Heparin quantification was performed using dimethylmethylene blue (DMMB, Sigma Aldrich, UK), with standards ranging between 0 and 25 μg mL^−1^ and an unconjugated SPION reference. The absorbance was measured at 525 nm using a SpectraMax M5 microplate reader (Molecular Devices, USA). The sulfated glycosaminoglycan content was normalized to the nanoparticle number, which was measured using a Nanosight (Ns300, Malvern Instruments, UK) equipped with Nanoparticle Tracking Analysis 3.0 software. Magnetic analysis was performed using a SQUID magnetometer (MPMS-7, Quantum Design, UK) on 1.5 mg mL^−1^ samples. These values were normalized by iron content, which was measured by digesting the nanoparticles in 2 N nitric acid (Sigma Aldrich, UK) at 40 °C for 24 h to ensure complete digestion and analyzing samples using inductively coupled plasma optical emission spectrometry (ICAP 6300 Duo, Thermo Fisher, USA).

The protein loading capacity of the glycosylated SPIONs was assessed by mixing avidin (Thermo Fisher, USA) with glycosylated and unconjugated SPIONs at room temperature for 10 min, and then centrifuging the sample at 20,817 g for 100 min. The concentration of avidin was detected using a Micro BCA™ Protein Assay Kit (Thermo Fisher, USA) following the manufacturer's protocol, using avidin standards between 0 and 20 μg mL^−1^. The loading capacity was determined by subtracting the initial loading mass of avidin with the mass of avidin detected in the supernatant.

### 2.2 Magnetic field alignment and simulation

Finite element magnetic modeling (FEMM, D. C. Meeker, Finite Element Method Magnetics, Version 4.2.) was used to characterize the magnetic field based on the dimensions and physical properties of the cylindrical N42 magnet (eMagnets, UK) used throughout the study (ø = 20 mm, height = 20 mm, HcB = 915 kA/m). All gradients were formed in a customized mold composed of two glass slides and a cut 2 mm silicone spacer (final well dimensions = 13 × 5 × 2 mm). 30 μL of solution or hydrogel precursor containing SPIONs were dispensed into the mold, followed by 60 μL of the same components minus the SPIONs. The magnetic field was subsequently applied from approximately 2 mm above the mold to redistribute the SPIONs into a gradient. To image the gradient formation process, gradients were formed in deionized water using a layer of 0.5 mg mL^−1^ SPIONs.

To examine the gradient forming capability in different hydrogels, a layer of 0.5 mg mL^−1^ SPIONs was used. After the formation of the gradient, the hydrogel precursor was gelled and the constructs were imaged using an Axio Observer inverted widefield microscope (Zeiss, Germany). Type VII-A Agarose (Sigma Aldrich, UK) was used at 1 wt% and gelled at room temperature. Geltrex (Thermo Fisher, USA) was used undiluted and gelled at 37 °C. Gellum gum (Sigma Aldrich, UK) was used at 0.75 wt% and crosslinked by the addition of a 0.06 wt% solution of calcium chloride. Gelatin (Sigma Aldrich, UK) was used at 5 wt% and gelled at 4 °C. To visualize the distribution of loaded protein, 10^11^ SPIONs were first loaded with 300 ng of fluorescein-labelled avidin (Thermo Fisher, USA) prior to gradient formation. Intensity plots were produced using the plot profile function on FIJI software using images acquired with an Axio Observer inverted widefield microscope (Zeiss, Germany). 1 wt% agarose was used to test the influence of viscosity on pattern formation, with the gradient forming process carried out in a temperature-controlled water bath (27, 30, 37 °C) and the resulting gels imaged using a desktop scanner. The viscosity of the agarose solutions at these temperatures was measured by rheological creep tests performed at 2 Pa stress over 5 min using an AR2000ex rheometer (TA Instruments, USA).

### 2.3 BMP-2 release kinetics

10^11^ glycosylated SPIONs loaded with 300 ng of BMP-2 (R&D System, USA) were cast into a 100 μL 1 wt% agarose hydrogel, to mimic the conditions used for osteochondral tissue engineering. The hydrogel was incubated in PBS with 0.02% of sodium azide (pH 7.4) (Sigma Aldrich, UK) at 37 °C for 28 days. 500 μL of PBS was removed at intervals and frozen at −20 °C, with 500 μL of fresh PBS added to the hydrogel to ensure that sink conditions were respected at all times. The concentration of released BMP-2 was then quantified using a BMP-2 specific ELISA kit (R&D System, USA), following the manufacturer's protocol. The absorbance was measured at 450 nm using a SpectraMax M5 plate reader. After the accumulated release was determined for each time point, the release profile was fitted by a Korsmeyer-Peppas model using KinetDs (version 3.0) [16]. The Korsmeyer-Peppas Model was developed to specifically model the release of molecules from a polymeric matrix, such as hydrogel network [17].

### 2.4 Stem cell culture and differentiation

hMSCs from three donors (Lonza, Switzerland) were expanded using Mesenchymal Stem Cell Growth Medium (MSCGM™, Lonza, Switzerland) in sterile culture at 37 °C/5% CO_2_. The culture medium was changed every 2–3 days, and hMSCs cultured up to passage 5. Differentiation was induced after encapsulating the hMSCs into agarose hydrogels at a cell density of 9 × 10^6^ hMSCs per mL. Osteogenic differentiation was triggered using high glucose Dulbecco's Modified Eagles Medium with pyruvate supplement (HG-DMEM 31966021, Thermo Fisher, UK) together with 10% (v/v) fetal bovine serum (FBS, Thermo Fisher, USA), 100 nM dexamethasone (Sigma Aldrich, UK), 50 μg mL^−1^ l-ascorbic acid 2-phosphate sesquimagnesium salt (l-ascorbic acid, Sigma Aldrich, UK) and 10 mM β-glycerophosphate (Sigma Aldrich, UK). Chondrogenic differentiation was triggered using HG-DMEM supplemented with 1X ITS^+^ (BD, USA), 100 nM dexamethasone, 50 μg mL^−1^ l-ascorbic acid, 50 μg mL^−1^ l-proline (Sigma Aldrich, UK) and 10 ng mL^−1^ transforming growth factor β3 (TGF-β3) (R&D System, USA). Osteochondral differentiation was triggered using HG-DMEM supplemented with 1X ITS^+^, 100 nM dexamethasone, 50 μg mL^−1^ l-ascorbic acid, 50 μg mL^−1^ l-proline, 2 mM β-glycerophosphate and 10 ng mL^−1^ TGF-β3. Where stated, BMP-2 was supplemented either in the medium (30 ng mL^−1^) or loaded together with glycosylated SPIONs. Unless otherwise specified, the BMP-2 concentration in the glycosylated SPIONs was 3 μg per mL of hydrogel.

### 2.5 Tissue engineering osteochondral gradients

10^11^ glycosylated SPIONs were incubated with 300 ng of BMP-2 at 4 °C for at least 5 h and then used to create a gradient in agarose, as described previously. hMSCs were included in both layers during the fabrication process, at a concentration of 9 × 10^6^ hMSCs per mL, and the final concentration of BMP-2 was 3 μg mL^−1^. The gradient hydrogel was transferred into a 24-well plate and cultured in 1 mL of osteochondral differentiation medium. The medium was changed after 2 h and the next day after seeding to remove the excess initial burst release BMP-2, and then three times a week for the remaining 27 days.

### 2.6 Gene expression analysis

Osteochondral tissue constructs engineered from three donors (N = 3, n = 3) were harvested and dissected into three equal pieces. The cartilage and bone ends were rinsed with PBS, homogenized using a TissueLyser II (Qiagen, Germany), and then stored at −80 °C. Total RNA was isolated using Trizol with Direct-zol™ RNA Kits, according to the manufacturer's protocol, and quantified using a NanoDrop 2000c (Thermo Fisher, UK). RNA was used to generate cDNA using a QuantiTect^®^ Reverse Transcription Kit (Qiagen, Germany), assuming a 1:1 conversion. Quantitative PCR was performed using 3 ng of cDNA, Taqman^®^ probes (Thermo Fisher, USA) and a StepOnePlus™ (Thermo Fisher, USA). The following probes were used: *RPL13A* (Hs04194366_g1), *ACAN* (Hs00153936_m1), *ALPL* (Hs01029144_m1), *COL1A1* (Hs00164004_m1), *COL2A1* (Hs00264051_m1), *COL10A1* (Hs00166657_m1), *RUNX2* (Hs01047973_m1), *SOX9* (Hs00165814_m1), *SP7* (Hs01866874_s1). The ΔΔCt method was used to compare expression at the bone region normalized to the cartilage region.

### 2.7 ALP and DNA assays

Osteochondral tissue constructs (N = 3, n = 3) were harvested and dissected, as previously described. The ALP activity and quantity of DNA was determined using a protocol modified from the literature [18]. The bone and cartilage ends of the tissue were homogenized separately using a TissueLyser II with ALP lysis buffer, consisting of 1 mM MgCl_2_ (Sigma Aldrich, UK), 20 μM ZnCl_2_ (Sigma Aldrich, UK) and 0.1% (w/v) octyl-β-glucopyranoside (Sigma Aldrich, UK) in 10 mM tris(hydroxymethyl)aminomethane buffer (pH 7.4) (Sigma Aldrich, UK) with the sample lysate immediately stored at −80 °C. To perform the assay, the samples were thawed on ice and then each sample was incubated with *p*-nitrophenol phosphate (Sigma Aldrich, UK) at 37 °C for 30 min. The reaction was terminated using 1 N NaOH and the absorbance measured at 405 nm using a SpectraMax M5 plate reader. A standard curve between 0 and 800 μM of *p*-nitrophenol phosphate was used to calculate the sample activity. An equivalent volume from the remainder of the sample was then used for DNA quantification using a PicoGreen™ assay (Thermo Fisher, UK), according to the manufacturer's protocol. The fluorescence was measured at 485/535 nm using a SpectraMax M5 plate reader. The ALP activity normalized to DNA quantity was compared between the bone and cartilage regions.

### 2.8 Histology and immunofluorescence staining

Osteochondral tissue constructs were harvested and washed three times in PBS, fixed with 4% (v/v) paraformaldehyde for 2 h at room temperature, washed a further three times in PBS and then paraffin embedded. 5 μm thick sections on Superfrost Plus slides (Thermo Scientific, UK) were deparaffinized using a 4 min incubation in Histo-Clear (National Diagnostics, USA) and hydrated using 2 min incubations in 100% ethanol, 70% ethanol and then deionized water. These sections were then stained using various protocols. For sulfated glycosaminoglycans, the sections were stained with Alcian Blue (pH 2.5) (Sigma Aldrich, UK) for 30 min, with a hematoxylin nuclear counterstain (Sigma Aldrich, UK). For calcium, Alizarin Red S (Abcam, UK) was used at a concentration of 2% (w/v) at pH 4.3. Alcian Blue and Alizarin Red S were performed on consecutive sections. The slides were mounted in Histomount (National Diagnostics, USA) and then imaged using a Zeiss Axio Observer Inverted Widefield Microscope. Images were superimposed after tiling.

For immunofluorescence staining, the sections were treated with proteinase K (Dako, USA) for antigen retrieval, and then blocked with 10% (v/v) donkey or goat serum. The samples were incubated overnight with goat anti type I collagen (Southern Biotech, USA) at 1/100 dilution, goat anti type II collagen (Southern Biotech, USA) at 1/20 dilution, rabbit anti osteopontin and rabbit anti type X collagen (Abcam, UK) at 1/100 dilution and goat and rabbit IgG negative controls (Abcam, UK) at 1/20 and 1/100 dilution. Excess primary antibody was then removed by three washes in PBS and sections were further incubated with secondary donkey anti goat or goat anti rabbit antibodies labelled with AlexaFluor 555 dye (Thermo Fisher, USA). DAPI (Thermo Fisher, USA) was used to counterstain the nucleus prior to mounting the sections with Vectashield Antifade Mounting Medium (Vector Lab, USA). Sections were imaged using a Zeiss Axio Observer Inverted Widefield Microscope.

### 2.9 Compression testing

Osteochondral tissue constructs were harvested, with uniform discs collected from the bone and cartilage regions using 2 mm biopsy punches (Miltex, USA). The biopsy dimensions were measured in the hydrated state using digital calipers for each construct, which were then soaked and tested in cOmplete™ Protease Inhibitor Cocktail (Roche, Switzerland). Unconfined compression testing was performed using a Bose Electroforce 3200 (BOSE, USA) equipped with a 250 g load cell. Compression stress relaxation tests were performed by imposing a square wave ramp for 12 steps of 5% strain with a 360 s hold using Wintest 7 (BOSE, USA). The instantaneous load was recorded at the beginning of each step and used to produce an instantaneous stress-strain data set, which was fitted with a bilinear model using Origin (OriginLab Corporation, USA). The instantaneous compressive modulus was then calculated using the low-strain linear region of the stress-strain curve and compared between the bone and cartilage regions.

### 2.10 Raman spectroscopy

Raman spectroscopic imaging was performed using a confocal Raman microscope (alpha300R+, WITec, Germany) equipped with a 532 nm laser and a x 20/0.4 NA objective lens (EC Epiplan, Zeiss, Germany). The scattered light was coupled to the spectrometer *via* a 100 μm fiber which functioned as the confocal pinhole. A 600 lines per mm grating spectrograph (UHTS 300, WITec, Germany) and a thermoelectrically cooled back-illuminated CCD camera (Newton DU970N-BV-353, Andor, UK) yielding a spectral resolution of ∼10 cm^−1^ (defined at full width at half maximum of mercury argon emission lines) were used to record spectra with 35 mW laser power at the sample. Full cross-sectional images with a 10 × 10 μm step-size were acquired with 0.5 s integration time while higher spatial resolution maps were recorded with 2 × 2 μm step size. Raman spectra were corrected for the instrument response of the system using a traceable Raman standard (STM-2245, National Institute of Standards and Technology). Engineered osteochondral tissue samples were prepared as previously described. After paraffin embedding and sectioning to 20 μm thickness on Superfrost Plus microscope slides, a standard dewaxing procedure was performed to remove confounding spectral signatures from the paraffin. Substrate signal was apparent in the acquired spectra in regions of low mineralization but did not affect the determination of mineral or cellular signatures. Full cross-sectional mineral profiles were calculated by integrating the measured HAP and TCP intensities (945 - 975 cm^−1^) at each position along the construct length. High spatial resolution maps correspond to HAP (ν_1_ PO_4_ at 962 cm^−1^), β−TCP (ν_1_ HPO_4_^2−^ at 948 cm^−1^), and cellular components of lipids and proteins (ν_as,s_ CH_2_ at 2850–3000 cm^−1^), which were experimentally validated in agreement with literature [19].

### 2.11 Cytotoxicity of glycosylated SPIONs

9.6 × 10^3^ hMSCs were seeded in a 96 well plate and left to adhere overnight before a 72 h incubation with 2 × 10^11^ or 10 × 10^11^ glycosylated SPIONs per mL of culture medium. The cytotoxicity of the glycosylated SPIONs was assessed using an alamarBlue assay (Thermo Fisher, USA), as per the manufacturer's instructions, with an incubation time of 3 h. The fluorescence was measured at 570/585 nm using an EnVision™ plate reader (Perkin Elmer, USA). The metabolic activity was normalized to an untreated control and averaged across three hMSC donors. hMSCs were also stained using a LIVE/DEAD™ assay (L3224, Thermo Fisher) and imaged using an IX51 Inverted microscope (Olympus, Japan).

### 2.12 Statistical analysis

Comparison between experimental groups was conducted using ordinary least-squares regression based on Generalized Linear Models in the statistical software R using the rms package. When appropriate, the data from the bone end of each individual construct was normalized to the cartilage end for direct pairwise analysis. The normalized data was then modelled as the dependent variable, and regression coefficients were calculated for independent variables. To account for different levels of response due to cell donor variability, generalized estimating equations were used (as previously described) to enable clustering of measurements obtained from constructs derived from a single donor [[20], [21], [22]]. The generated regression coefficients were compared. Specifically for this analysis, the relative changes of interest were the gene expression, enzyme activity, or mechanical properties (categorical). These descriptors were included as independent variables. Heteroscedasticity in the data set was addressed by using the robust covariance function created in the rms package (“robcov”) to adjust the standard errors. Finally, to compare groups, a Welch's *t*-test (for two group comparisons) or a one sample Student's t-test was performed on the generated GLM regression coefficients from the developed regression model, which were represented with *p*-values. All hypothesis tests were considered one-tailed based on the hypothesis of increased values in the bone end of the construct relative to the cartilage end, with exceptions for cell viability and expression levels of chondrogenic genes (*ACAN*, *COL2A1*, and *SOX9*).

## 3 Results and discussion

In order to generate biochemical gradients, we glycosylated 20 nm SPIONs with a protein-binding corona of heparin. Briefly, we used a carbodiimide-based coupling to conjugate the carboxylate groups of heparin to primary amines present on the nanoparticle surface. We then characterized the glycosylated SPIONs, which were colloidally stable, using dynamic light scattering in order to determine the size and surface charge potential (Table 1). The hydrodynamic diameter increased from 48.6 ± 0.2 nm for the bare nanoparticles to 72.3 ± 0.9 nm after glycosylation, which was attributed to the steric bulk of the heparin corona. The glycosylated SPIONs exhibited a zeta potential of −54.4 ± 1.7 mV compared to −4.7 ± 0.6 mV for the unconjugated nanoparticles, which was further evidence for the binding of heparin, a highly anionic polysaccharide. We assessed the magnetic properties of the glycosylated SPIONS using superconducting quantum interference device (SQUID) magnetometry correlated with inductively coupled plasma optical emission spectroscopy (ICP-OES). The glycosylated SPIONs exhibited a magnetic susceptibility of 5.4 ± 0.0 × 10^−1^ emu g^−1^ Oe^−1^ and a saturation of 53.6 ± 0.4 emu g^−1^, values that were very similar to the unconjugated nanoparticles (5.2 ± 0.0 × 10^−1^ emu g^−1^ Oe^−1^, 54.9 ± 0.3 emu g^−1^) (Table 1).

**Table 1:** Biophysical characterization of glycosylated SPIONs (mean ± standard deviation).

	Scattering Analysis		Magnetic Characterization		Biochemical Profile	
	Hydrodynamic Diameter/nm	Zeta Potential/mV	Magnetic Susceptibility/×10^−1^ emu g^−1^ Oe^−1^	Magnetic Saturation/emu g^−1^	Degree of Glycosylation/ng per 10^9^ SPIONs	Protein Sequestered/ng per 10^9^ SPIONs
Unconjugated SPIONs	48.6 (±0.2)	−4.7 (±0.6)	5.2 (±0.0)	54.9 (±0.3)	N/A	1.5 (±0.3)
Glycosylated SPIONs	72.3 (±0.9)	−54.4 (±1.7)	5.4 (±0.0)	53.6 (±0.4)	4.2 (±0.5)	4.7 (±0.2)

Next, we measured the degree of glycosylation using nanoparticle tracking analysis and a dimethylmethylene blue (DMMB) assay (Supplementary Figure 1 and Table 1). These results revealed 4.2 ng of heparin per 10^9^ nanoparticles, a value that corresponded to approximately 140 heparin chains bound to the surface of each nanoparticle. The high density of carboxylate groups on heparin provides an anionic network that can be used to sequester many soluble proteins. For example, our protein of interest, bone morphogenetic protein 2, exhibits highly basic N-terminal peptide domains that confer a high binding affinity with heparin (kD ≈ 20 nM) [23]. To assess the sequestration of the glycosylated SPIONs, we used avidin as a model protein, due to its similar isoelectric point, comparable dissociation constant (kD ≈ 160 nM) and the fact that it is commonly used as an analogue for studying the interaction between BMP-2 and heparin [24,25]. A bicinchoninic acid assay of avidin revealed an average of 4.7 ± 0.2 ng of avidin incorporated per 10^9^ nanoparticles, approximately four times greater than observed for the unconjugated SPIONs, and equivalent to approximately 40 sequestered proteins per nanoparticle. This loading capacity offered the opportunity to deliver more than 400 ng of growth factors, a quantity sufficient to influence cell fate, using only 10^11^ nanoparticles [26]. Importantly, the glycosylated SPIONs did not significantly affect the viability of hMSCs, as demonstrated using alamarBlue and LIVE-DEAD™ assays performed after a 72-h exposure to glycosylated SPIONS (10^12^ nanoparticles per mL) (Supplementary Figure 2).

**Fig. 2:**
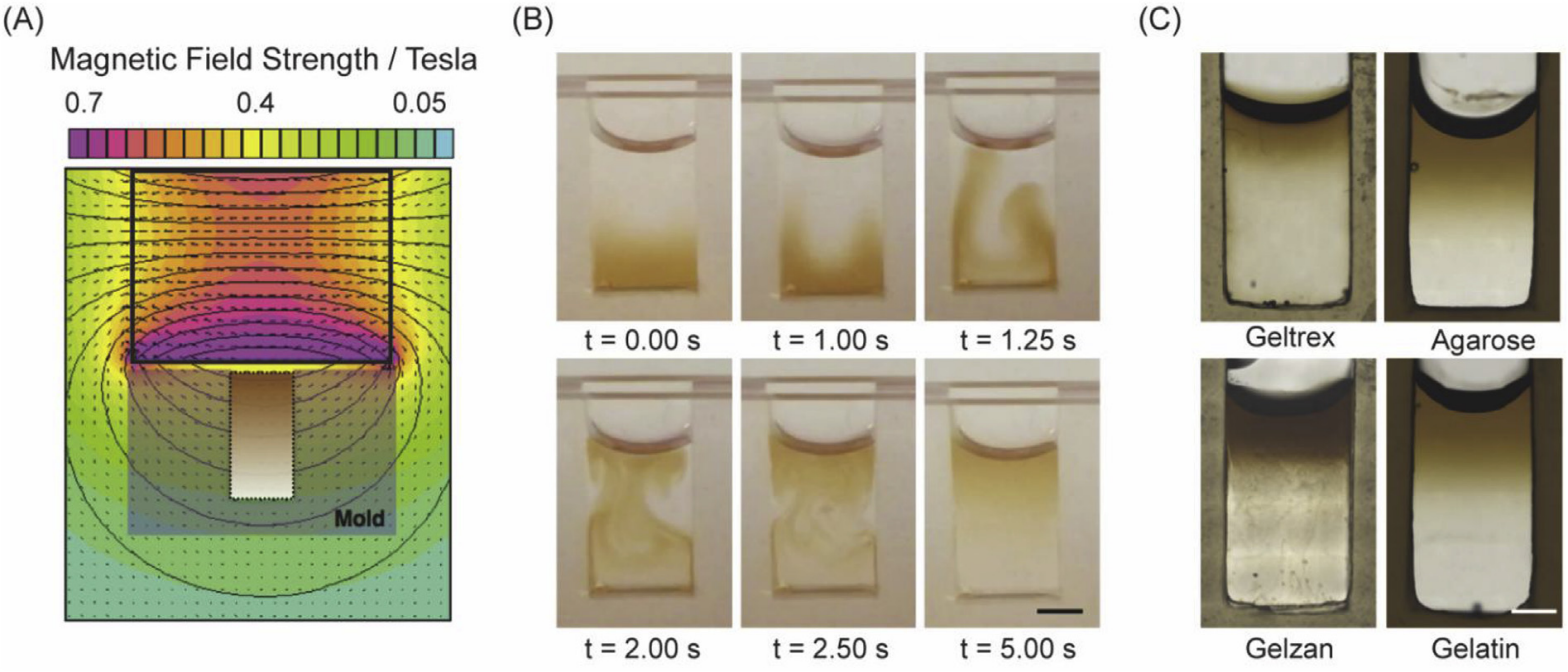
Magnetic field alignment of SPIONs. (A) Finite element modeling of the magnetic field strength and distribution. (B) Time-lapse images showing the magnetic field alignment of SPIONs (brown) in water, with a clear transition from a biphasic stack to a smooth gradient. (C) Bright field microscopy images showing gradients of SPIONs (brown) immobilized in different biomaterials. Scale bar in all images = 2 mm. (For interpretation of the references to colour in this figure legend, the reader is referred to the Web version of this article.)

Next, we investigated the nanoparticle response to externally-applied magnetic fields in aqueous solution and different hydrogels commonly used for *in vitro* tissue engineering. Here, we used finite element magnetic modeling to characterize the magnetic field strength and distribution (Fig. 2A). This map enabled us to define the relative positions of the magnet and nanoparticle solution, in order to expose the SPIONs to a well-defined field gradient (from 0.1 to 0.4 T) (Supplementary Figure 3). We initially used these field parameters to attract SPIONs dispersed in a monophasic suspension, however, this process took several hours (>16 h) to reach completion and produced a thin layer of aggregated SPIONs rather than a gradient transition. We resolved this issue by starting with a two-phase system, in which nanoparticle-free solution was stacked on top of a suspension of nanoparticles. Applying a magnetic field from above resulted in a rapid redistribution of the two layers to generate a smooth nanoparticle gradient (<5 s) (Fig. 2B). Continued exposure to the magnetic field eventually resulted in the SPIONs forming dense aggregates close to the magnet. This transition was a slow process (>10 h), which afforded ample time to capture the system in a non-equilibrium state by encapsulating the nanoparticle gradient in a hydrogel. Indeed, this process appeared to be largely independent of the material type or crosslinking mechanism, however, we did identify three key characteristics that favored gradient formation. First, the SPIONs should be colloidally stable in the precursor solution; which was not the case for certain gels at high weight fraction (*e.g.* > 5 wt% agarose). Second, the precursor solution should not inhibit SPION movement through viscous drag; no gradient formation was observed in 1% agarose gels maintained as a highly viscous solution at 27 °C (Supplementary Figure 4). Third, the crosslinking mechanism should be reasonably rapid (<1 h), and preferably triggered, in order to capture the SPIONs in a non-equilibrium gradient distribution. These characteristics are exhibited by the majority of hydrogels used for tissue engineering. Indeed, we successfully demonstrated the formation of SPION gradients in a range of biomaterials, including gelatin, Geltrex™, Gelzan™ and agarose (Fig. 2C). Importantly, we could use this system to generate protein gradients in biomaterials, as demonstrated by fluorescent microscopy of an agarose hydrogel patterned with glycosylated SPIONs containing fluorescently-tagged avidin (Supplementary Figure 5A). The protein cargo was well co-localized with the nanoparticles, and importantly, the magnetic field alignment produced a much smoother transition than a biphasic stacking approach, which resulted in a much sharper boundary at the layer interface (Supplementary Figure 5B).

**Fig. 3:**
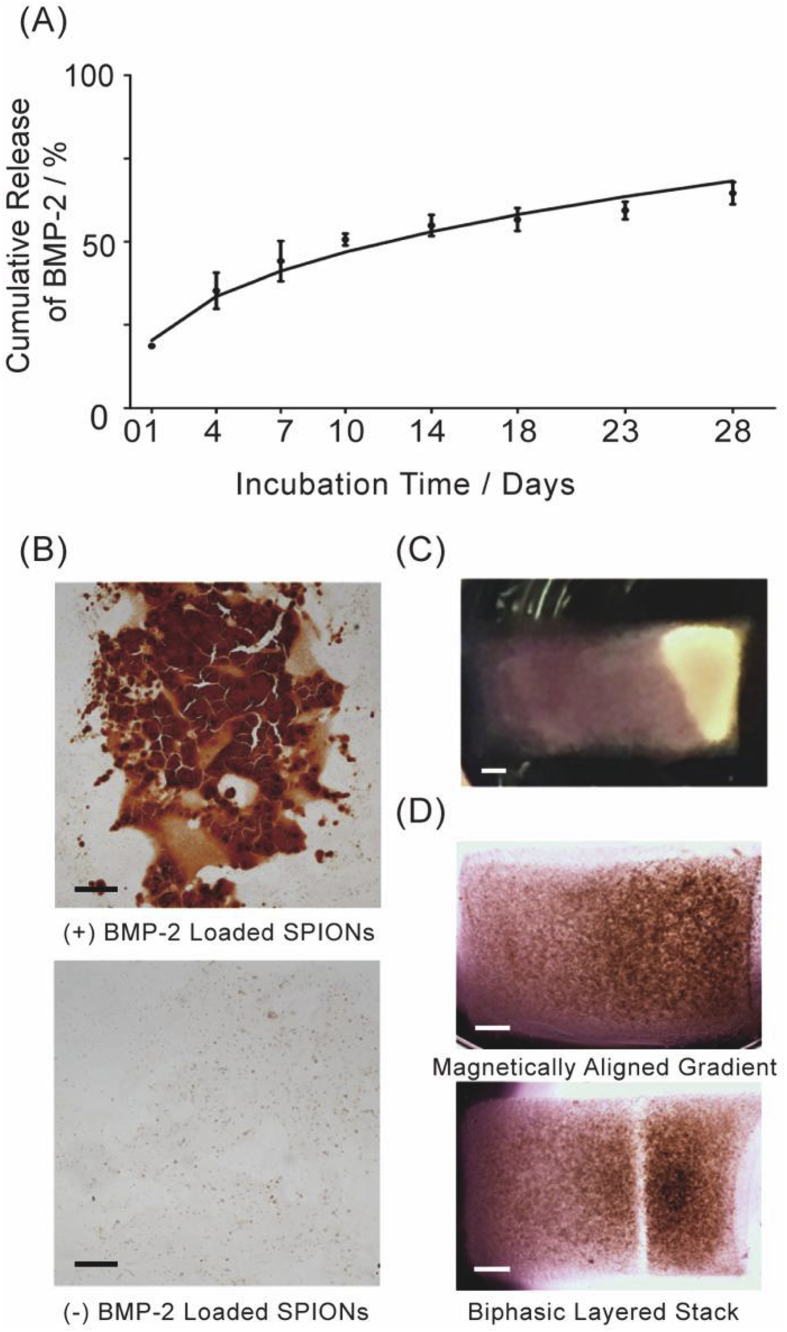
BMP-2 release and action in agarose hydrogels. (A) An ELISA was used to detect the release of BMP-2 from glycosylated SPIONs immobilized in 1 wt% agarose, over a period of 28 days. (mean ± S.D., n = 3). The BMP-2 release profile from agarose to the surrounding medium was fitted using the Korsmeyer-Peppas model (R^2^ = 0.97). (B) Alizarin Red S staining in tissue engineered hMSC-laden agarose constructs with or without glycosylated SPIONs loaded with BMP-2. Extensive calcium deposition (red) was observed in the hydrogel with BMP-2 present, with no staining in the system without BMP-2. Scale bars = 200 μm. (C) Macroscopic view of a representative osteochondral tissue construct engineered using agarose hydrogels patterned with a SPION gradient of BMP-2. An opaque mineralized region can be clearly observed at the bone end of the tissue construct. Scale bars = 1 mm (D) For the gradient constructs, bright field microscopy showed a smooth transition from the cartilage region to the bone region. A control system using a biphasic layered stack, rather than a gradient, exhibited a clear margin between the two regions of the tissue. Scale bars = 1 mm. (For interpretation of the references to colour in this figure legend, the reader is referred to the Web version of this article.)

We applied this hydrogel patterning approach to the complex and clinically-relevant challenge of osteochondral tissue engineering. Native osteochondral tissue possesses a structural transition from hyaline cartilage to the underlying subchondral bone that is generated in the developmental process of endochondral ossification. A key biochemical cue present during tissue development is the pro-osteogenic growth factor BMP-2. Gradients of BMP-2 in the growth plate play an important role in spatially regulating chondrocyte proliferation and hypertrophic differentiation *in vivo* [3,27], while *in vitro* studies have shown that BMP-2 can trigger bone formation in a concentration-dependent manner [28]. Like many cationic growth factors, BMP-2 exhibits a high affinity for heparin (kD = 20 nM) [23], which has been used in tissue engineering systems to guard against degradation and provide sustained release and stimulation [29,30]. To test whether our system could be used to release growth factors in a biomaterial system, we immobilized BMP-2 loaded glycosylated SPIONs in agarose hydrogels and then monitored the release into solution using an enzyme-linked immunosorbent assay (ELISA). This analysis revealed a sustained diffusion-driven release (Korsmeyer-Peppas model fitting, R^2^ = 0.97) of BMP-2 over 28 days, with 20% released after the first 24 h (Fig. 3A). It should be noted that these measurements do not account for any possible denaturation of BMP-2. Accordingly, we sought to test the functional activity of the loaded glycosylated SPIONs in a tissue engineering set up.

Here, we intend to use magnetic alignment to present glycosylated SPIONs loaded with BMP-2 as a gradient across a hMSC-laden agarose hydrogel. The subsequent slow release of active BMP-2 over 28 days should stimulate hMSC osteogenesis and tissue mineralization predominantly at one end of the engineered construct [31]. To ensure cartilage formation at the other end of the tissue, we included TGF-β3 alongside insulin-transferrin-selenium, ascorbic acid, dexamethasone and β-glycerophosphate in an optimized osteochondral differentiation medium based on similar compositions reported in the literature [13,32,33]. We used β-glycerophosphate at a concentration of 2 mM, a level that can provide a source of phosphate without initiating osteogenesis or nonspecific mineral precipitation [34]. Indeed, we showed that this osteochondral medium could support either osteogenic or chondrogenic differentiation of hMSCs in agarose, depending on the presence or absence of BMP-2 in the culture medium (Supplementary Figure 6). More importantly, the osteochondral medium supported tissue mineralization in a 28-day culture of hMSCs in agarose with BMP-2 delivered exclusively *via* glycosylated SPIONs (Supplementary Figure 7). Indeed, the total level of BMP-2 loaded into the glycosylated SPIONs was often too high, with a large excess of unconsumed growth factor capable of diffusing out of the hydrogel and stimulating osteogenesis in adjacent, nanoparticle-free constructs. We addressed this issue by optimizing the level of BMP-2 in the glycosylated SPIONs to identify a growth factor concentration (3 μg mL^−1^) capable of producing only local mineralization (Fig. 3B and Supplementary Figure 8).

Having optimized the gradient formation, growth factor release, cell viability and differentiation for osteochondral tissue engineering, we assembled the final system with BMP-2 loaded glycosylated SPIONs magnetically-aligned within a hMSC-laden agarose hydrogel. Over 28 days of differentiation, the vast majority of the tissue constructs developed striking white opacity at the bone end of the constructs (Fig. 3C). It should be noted that approximately 10% of the engineered constructs failed to produce any visible mineralization, and these were excluded from further study. In all cases, however, the gradient hydrogels produced tissue constructs that were structurally robust, with no macroscopic defects observed between the bone and cartilage ends of the tissue. This was in stark contrast with osteochondral tissue engineered from stacked, biphasic constructs, which produced a distinct 200 μm margin between the two ends of the tissue (Fig. 3D). Further investigation revealed that these margins were formed prior to tissue culture, with the biphasic interface appearing to exclude hMSCs (Supplementary Figure 9). The exact mechanism underpinning this observation is unknown, but nevertheless, the ability to avoid such tissue defects represents a major advantage for our gradient biomaterial over simple biphasic systems.

**Fig. 4:**
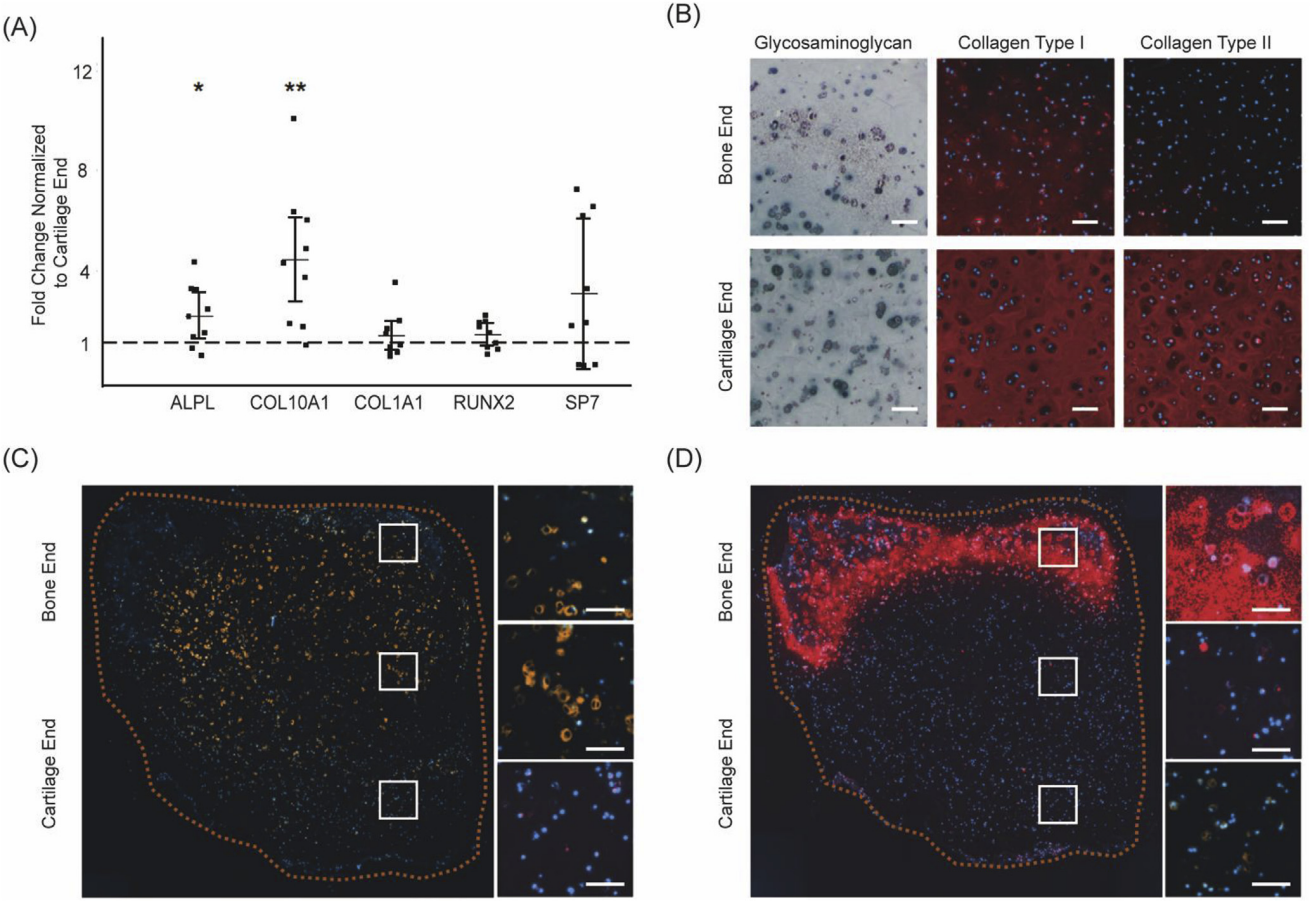
Gene and protein expression in osteochondral tissue engineering. (A) Gene expression of five osteogenic genes at the bone region of the tissue, compared to the cartilage region. Comparison of differences were made using a one sample *t*-test after heteroscedasticity in the dataset was addressed (mean ± 95% confidence intervals, N = 3, n = 3) where p < 0.05 (*), p < 0.01 (**). (B) Histological and immunofluorescence staining of key extracellular matrix proteins present in cartilage and bone revealed deposition of sulfated glycosaminoglycans (blue) and type I and II collagen (red). Scale bars = 200 μm. (C) Immunofluorescence staining of the hypertrophic protein type X collagen (orange), and (D) the key mineralization protein osteopontin (red), which were present specifically at the bone end of the tissue. Scale bars = 200 μm. (For interpretation of the references to colour in this figure legend, the reader is referred to the Web version of this article.)

Alongside these macroscopic observations, we characterized the osteochondral tissue gradient constructs after 28 days of culture using gene expression analysis, histology and immunostaining. Gene expression analysis using quantitative polymerase chain reaction (qPCR) revealed significant upregulation in the mRNA of both chondrogenic genes (*COL2A1*, *SOX9*, *ACAN*) and osteogenic genes (*SP7*, *RUNX2*, *COL1A1*, *ALPL*, *COL10A1)*, compared to undifferentiated hMSCs at day 0 (Supplementary Figure 10). The observed upregulation indicated a positive effect of both the media-supplemented TGF-β3 and the SPION-loaded BMP-2. More importantly, we observed a higher expression of osteogenic genes in the bone region compared to the cartilage region (N = 3, n = 3) (Fig. 4A). Significantly upregulated genes included *ALPL* and *COL10A1*, which are responsible for the expression of alkaline phosphatase and type X collagen, respectively. Importantly, we observed no significant differences between the bone and cartilage ends of the construct for any of the chondrogenic genes screened (Supplementary Figure 11). This gene expression analysis strongly indicated that the soluble TGF-β3 acted globally across the tissue, while the magnetically-aligned gradient of BMP-2 influenced the osteogenic differentiation of hMSCs in a spatially-defined manner.

Furthermore, we showed that these differences in gene expression could spatially influence matrix composition and structure in the tissue constructs. Alcian Blue staining for sulfated glycosaminoglycans and immunofluorescence staining for type I and II collagen revealed extensive extracellular matrix deposition, with cells located in well-defined lacunae (Fig. 4B). The presence of these components was expected; glycosaminoglycan and type II collagen are the two major components of physiological hyaline cartilage, while type I collagen is commonly expressed in engineered cartilage constructs, particularly those grown using hMSCs [35]. Although we observed no spatial differences in the gene expression of *COL1A1* and *COL2A1*, the immunostaining appeared to show a higher quantity of both type I and II collagen at the cartilage end of the tissue. The discrepancy between the mRNA and protein levels can be attributed to the fact that gene expression and matrix remodeling are dynamic processes that are not always correlated in an end-point tissue analysis [36]. Interestingly, we observed type X collagen predominantly at the interface between the bone and cartilage regions, which indicated a zone of hypertrophic chondrocytes [37] (Fig. 4C). Furthermore, osteopontin, a key marker of osteogenesis and biomineralization, was present exclusively in the bone region of the tissue (Fig. 4D).

**Fig. 5:**
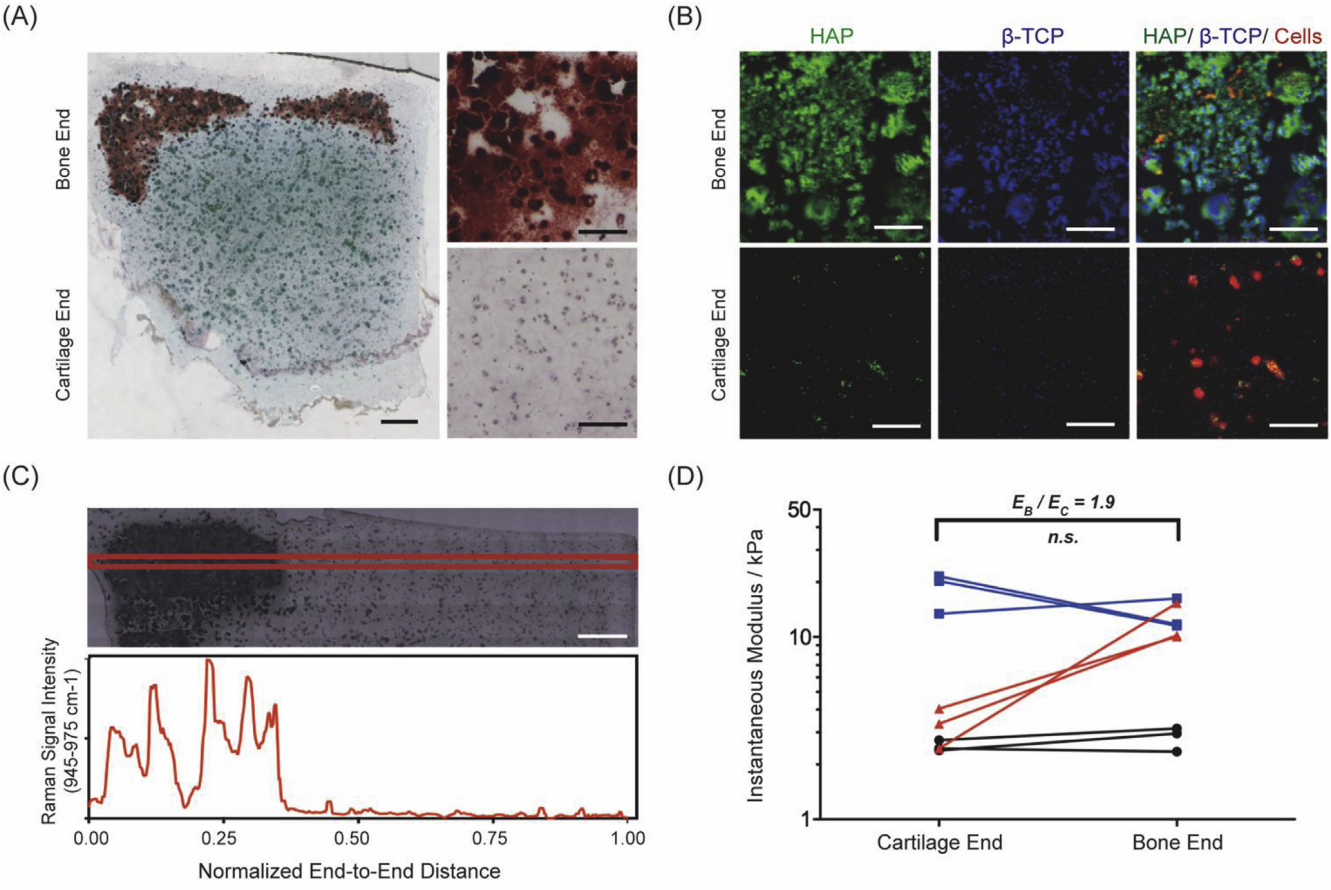
Mineralization and mechanical analysis of engineered osteochondral tissue. (A) Alizarin Red S staining of calcium deposits (red) and Alcian Blue staining of sulfated glycosaminoglycan (blue) in engineered osteochondral constructs showed mineralization specifically at the bone end of the tissue. Scale bars = 500 μm. (B) Raman microscopy of HAP (green), β-TCP (blue) and cells (red) also showed mineralization specifically in the bone region. Scale bars = 100 μm. (C) Profile of Raman intensity corresponding to integrated signal from HAP and β-TCP (945 - 975 cm^−1^) across the length of the osteochondral tissue construct, with a bright field microscopy image for reference. Scale bar = 500 μm. (D) Unconfined compression testing of the engineered osteochondral constructs showing the instantaneous modulus and the pairwise comparison between tissue constructs engineered from three different hMSC donors (denoted by red, black and blue markers). Comparison of differences were made using a one sample *t*-test after heteroscedasticity in the dataset was addressed (mean ± 95% confidence intervals, N = 3, n = 3) where p < 0.05 (*). (For interpretation of the references to colour in this figure legend, the reader is referred to the Web version of this article.)

This spatially-controlled osteogenic response was further corroborated using an alkaline phosphatase assay, which revealed a higher enzymatic activity in the bone region, compared to the cartilage region **(**Supplementary Figure 12**).** Alkaline phosphatase catalyzes the hydrolysis of organic phosphates into inorganic phosphate ions, which are used in tissue mineralization [38]. Accordingly, we further investigated the mineral formation in the engineered tissue. Alizarin Red S staining revealed the bone region to be comprised of dense calcium-rich nodules (Fig. 5A), while Raman microscopy identified the presence of two distinct calcium phosphate morphologies: hydroxyapatite (HAP) and β-tricalcium phosphate (β-TCP) (Fig. 5B). HAP is a non-degradable and mechanically-robust mineral, while β-TCP is a bioresorbable salt with osteoinductive degradation products; the presence of both morphologies is desirable during bone formation [39,40]. Importantly, in both the Alizarin Red S staining and the Raman mapping, we detected negligible calcium phosphate in the cartilage region. Indeed, by integrating the Raman contributions from β-TCP and HAP, we were able to detect a sharp transition in mineral content from bone to cartilage that resembles the tidemark of the osteochondral interface [41] (Fig. 5C, Supplementary Figure 13). This observation was consistent with both the osteopontin immunofluorescence staining and the macroscopic images of the engineered tissue. Finally, we assessed the mechanical properties of the osteochondral constructs using an unconfined compression test (N = 3). The instantaneous stress-strain response of the bone and cartilage regions revealed a higher instantaneous modulus at the bone end than at the cartilage end in one of the biological replicates (Fig. 5D and Supplementary Figure 14). This observation could be attributed to differences in matrix composition, such as the presence of mineral in the bone region [42].

Overall, our results indicate that BMP-2 signaling gradients patterned using glycosylated SPIONs can spatially influence osteogenic gene expression to generate robust osteochondral constructs exhibiting low-stiffness cartilage and higher stiffness mineralized bone. Moreover, the histology, immunofluorescence staining and Raman microscopy provide a detailed picture of the engineered osteochondral tissue. In particular, we can define three major zones; a mineralized *bone region* that sharply switches into an *intermediate region* of hypertrophic chondrocytes, which then smoothly transitions into a collagen-rich *cartilage region*. To the best of our knowledge, such complexity has not been demonstrated using biphasic hydrogel systems. Interestingly, the structural complexity of our tissue, most notably the sharp transition between bone and cartilage followed by the region of hypertrophic chondrocytes, was generated from an initially smooth concentration gradient of BMP-2. These emergent structural properties are fascinating considering the developmental biology of the osteochondral interface, in which concentration gradients of osteoinductive factors produce a sharp transition (tidemark) that separates the subchondral bone/calcified cartilage from hyaline cartilage. We hypothesize that a threshold level of BMP-2 may be required to initiate and sustain mineralization of our tissue, which may in turn produce a positive feedback loop whereby the deposited mineral (HAP or resorbed β-TCP) act to further stimulate osteogenesis. Further work is required to test this theory, information that will provide a sound mechanistic basis for engineering of osteochondral constructs and other structurally complex tissues using our gradient technology. Additional work is also required for translation of this technology; in particular, the overall mechanical properties of our tissue construct must be raised to a level that is compatible with *in vivo* transplantation. More generally, clinical translation of our gradient biomaterials will require the use of materials, cells and media approved by the Food and Drug Administration (FDA) and other relevant international agencies, and the fabrication procedures to be brought in line with good manufacturing and clinical practice (GMP/GCP) guidelines.

## 4 Conclusions

Any robust and relevant engineered graft must recapture the complex hierarchical microstructure of the physiological tissue. In this report, we introduce a novel platform based on magnetic field alignment that can be used to rapidly produce smooth biochemical gradients for osteochondral tissue engineering. Specifically, the osteogenic growth factor BMP-2 was sequestered into glycosylated SPIONs, which were magnetically patterned across an agarose hydrogel laden with hMSCs. Diffusion of BMP-2 from the glycosylated corona provided spatially-directed osteogenesis to generate robust osteochondral tissue constructs. The initial gradient of BMP-2 gave rise to emergent structural features that highly resembled the osteochondral interface: mineralized bone transitioning to cartilage *via* a region of hypertrophic chondrocytes. The osteochondral constructs exhibited distinct cell phenotypes (chondrocytes and osteoblasts), protein expression (presence of osteopontin, alkaline phosphatase activity) and tissue mineralization (presence of β-TCP and HAP mineral). Taken together, the gene expression, biochemical profile, matrix distribution and Raman microscopy suggested that the interplay between the released BMP-2 and the solution factors (TGF-β3, β-glycerophosphate) were at a point where the tissue calcification could be achieved in a local fashion governed entirely by the patterned gradient. This technology offers great versatility and could be readily tailored to other growth factors and hydrogel systems, offering new opportunities for a range of interfacial tissue engineering challenges.

## Author contributions

The study was designed by C.L., J.P.K.A. and S.C.C., with C.L. performing the majority of experimental work. C.L., J.P.K.A. and S.C.C. performed preliminary feasibility studies, I.J.P. performed all Raman spectroscopy experiments and assisted with the statistical analysis. S.W.K., J.L.P. and A.C.M. assisted with mechanical characterization and J.L.P. also provided guidance on histology and ALP assays. M.M.S. supervised the project and C.L., J.P.K.A. and M.M.S. wrote the paper, with contributions from all authors.
