# Supplementary material for: Glycosylated superparamagnetic nanoparticle gradients for osteochondral tissue engineering
Source: Biomaterials. 2018 Sep;176:24–33. doi: 10.1016/j.biomaterials.2018.05.029 (PMC6018621; doi:10.1016/j.biomaterials.2018.05.029)
Supplement: Multimedia component 1 [file mmc1.docx]

**Glycosylated Superparamagnetic Nanoparticle Gradients for Osteochondral Tissue Engineering**

Chunching Li,^1^ James PK Armstrong,^1^ Isaac J Pence,^1^ Worrapong Kit-Anan,^1^ Jennifer L Puetzer,^1^ Sara Correia Carreira,^2^ Axel C Moore,^1^ Molly M Stevens^1*^

1. Department of Materials, Department of Bioengineering and Institute of Biomedical Engineering, Imperial College London, Prince Consort Road, London, SW7 2AZ, United Kingdom.
2. H. H. Wills Physics Laboratory, University of Bristol, Tyndall Avenue, Bristol, BS8 1TL, United Kingdom.

- Corresponding author email address: [m.stevens@imperial.ac.uk](mailto:m.stevens@imperial.ac.uk)


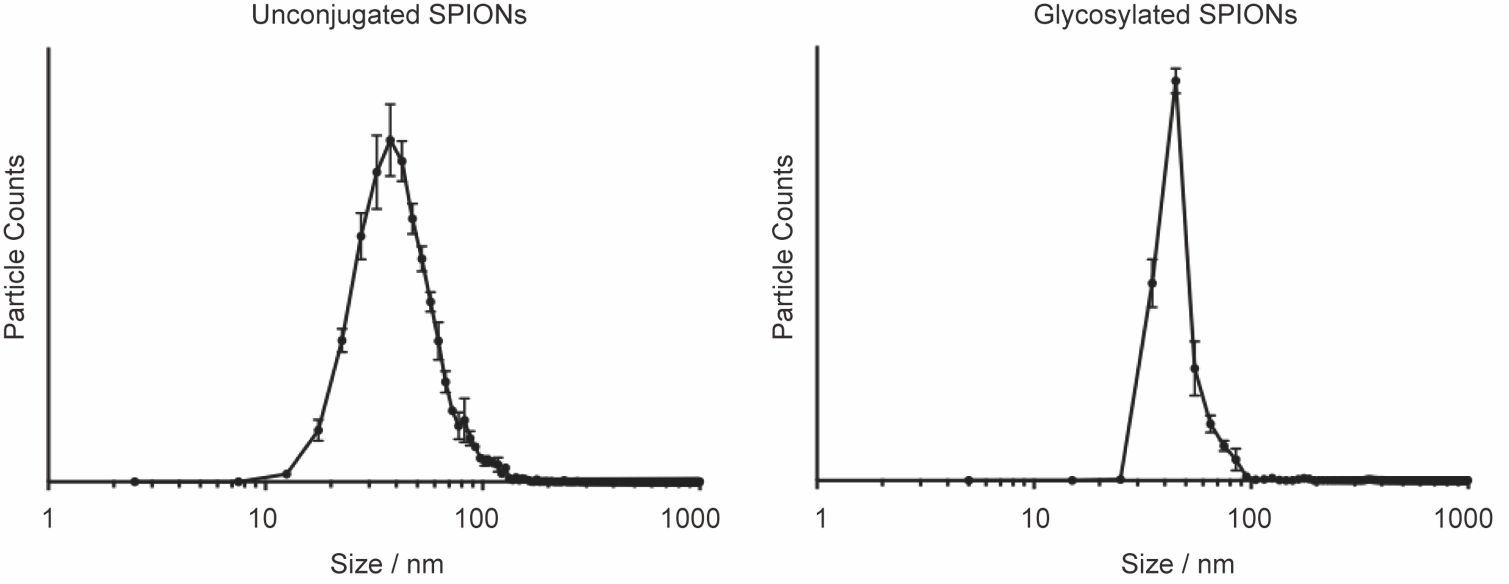


**Supplementary Figure 1.**

**Nanoparticle tracking analysis of unconjugated and glycosylated SPIONs.**

**
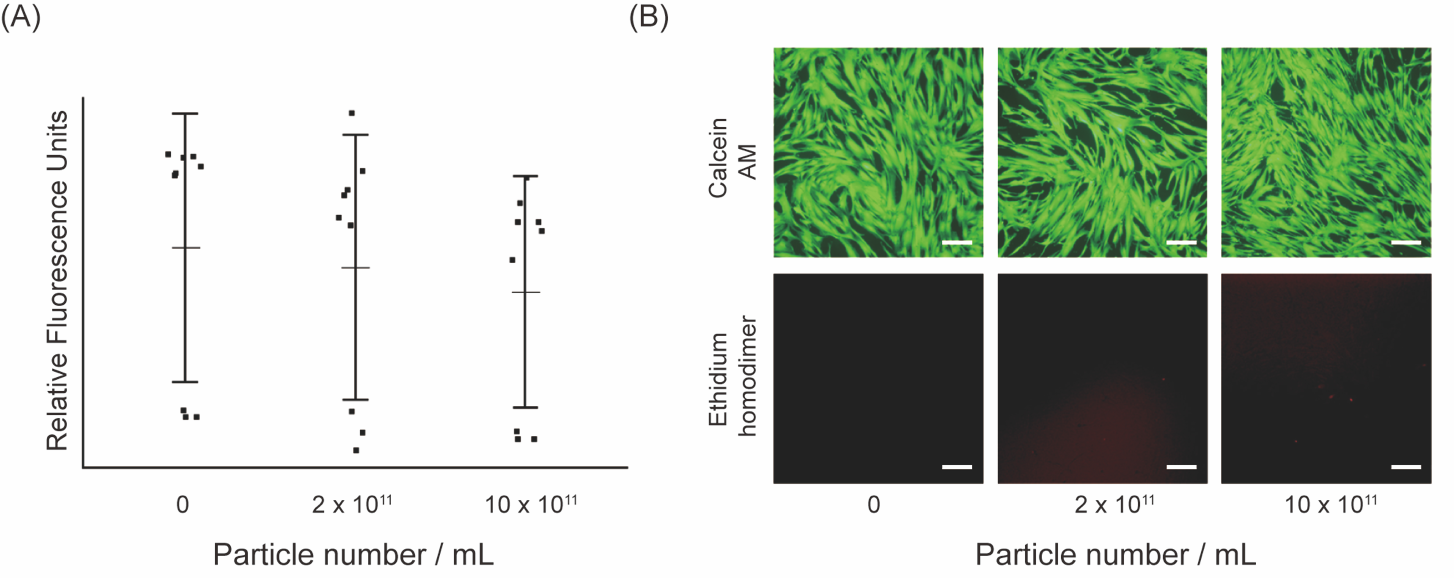
**

**Supplementary Figure 2.**

**Viability analysis of glycosylated SPIONs. (A) An alamarBlue assay of the effect of glycosylated SPIONs upon hMSCs, with values representing the metabolic activity of hMSCs exposed to 0, 2 x 10^11^ or 10 x 10^11^ glycosylated SPIONs. (mean ± 95% confidence intervals, N = 3, n = 3). (B) LIVE/DEAD^TM^ staining of hMSCs treated with glycosylated SPIONs showing a large number of viable cells (green, calcein AM) and negligible staining for dead cells (red, ethidium homodimer). Scale bars = 200 µm.**


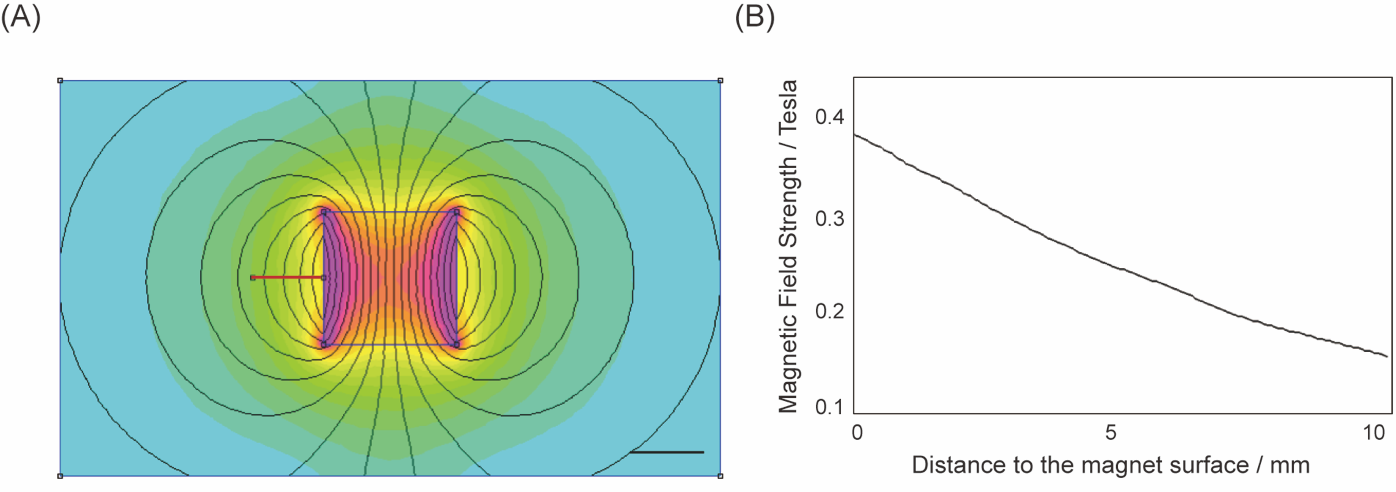


**Supplementary Figure 3.**

**(A) Finite element magnetic modeling of the externally applied magnetic field. (B) The magnetic field strength plotted as a function of distance from the magnet surface revealed a smooth gradient across the exposed SPION solution. Scale bar = 1 cm.**

**
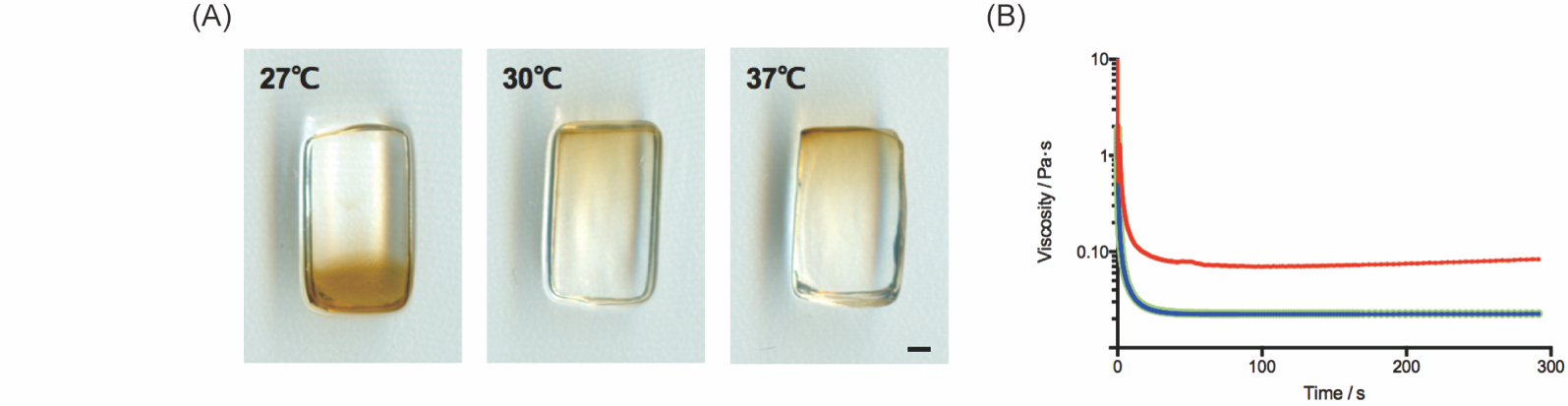
**

**Supplementary Figure 4.**

**To test the effect of viscosity upon gradient formation, the gradient formation process was tested using 1 wt% agarose solutions at different temperatures. The increased viscosity at 27°C appeared to inhibit the gradient formation process. Scale bar = 1 mm. (B) Rheological creep tests confirmed that the 1 wt% agarose solution at 27°C (red trace, 0.07 Pa s) was of higher viscosity than the 1 wt% agarose solutions at 30°C (blue trace, 0.02 Pa s) and 37°C (green trace, 0.02 Pa s). The viscosity was evaluated using the plateau region from 100-300 s of duplicate measurements from separate agarose solutions.**


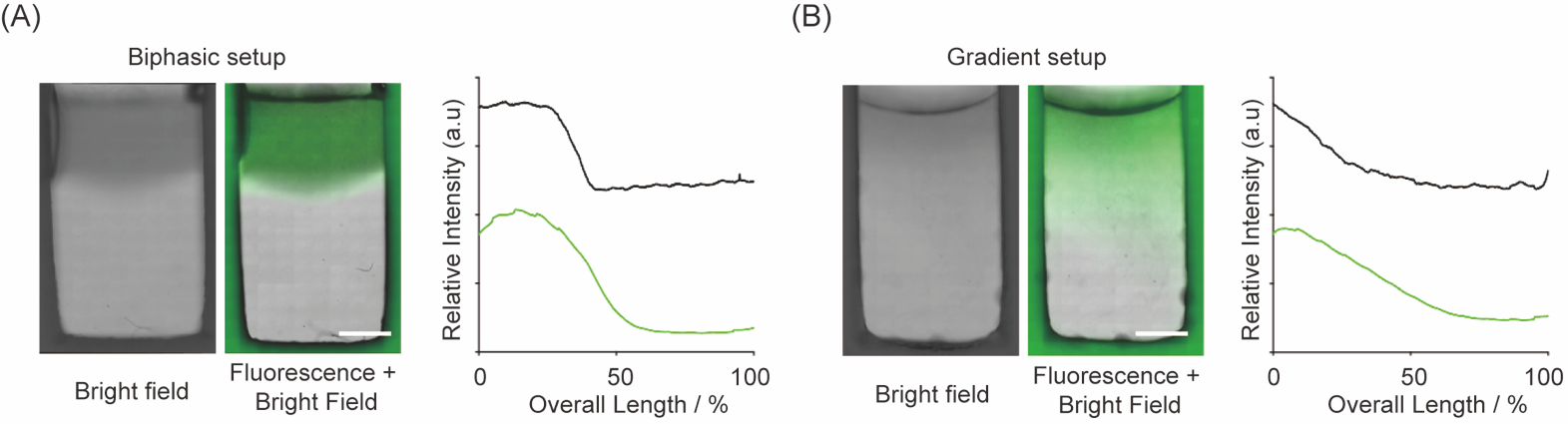


**Supplementary Figure 5.**

**The distribution of loaded fluorescently-tagged avidin (green) in agarose was visualized using fluorescence microscopy. Corresponding bright field images revealed the distribution of glycosylated SPIONs (dark grey). (A) Biphasic stacking of hydrogel layers resulted in a much sharper boundary at the layer interface compared to (B) magnetic field alignment, which produced a smooth gradient. Scale bars = 2 mm.**


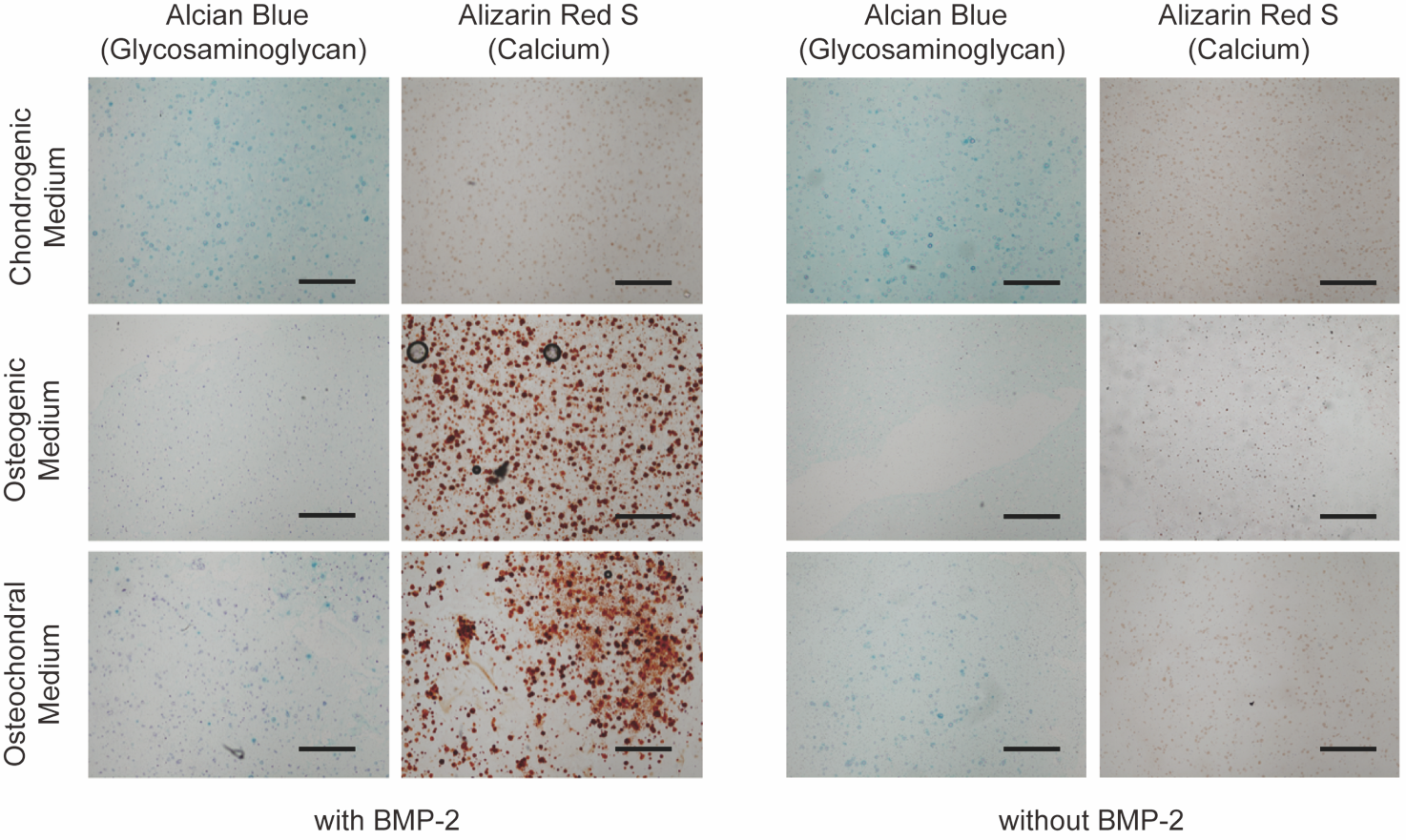


**Supplementary Figure 6.**

**Effect of medium composition and BMP-2 upon osteochondral tissue engineering. Tissue constructs were engineered for 28 days using hMSC-laden agarose hydrogels. Alizarin Red S staining showed deposition of calcium mineral (red) when exposed to BMP-2 and cultured in osteogenic and osteochondral medium. Alcian Blue staining showed glycosaminoglycan (blue) in chondrogenic and osteochondral medium, either in the presence or absence of BMP-2. Scale bars = 500 µm.**


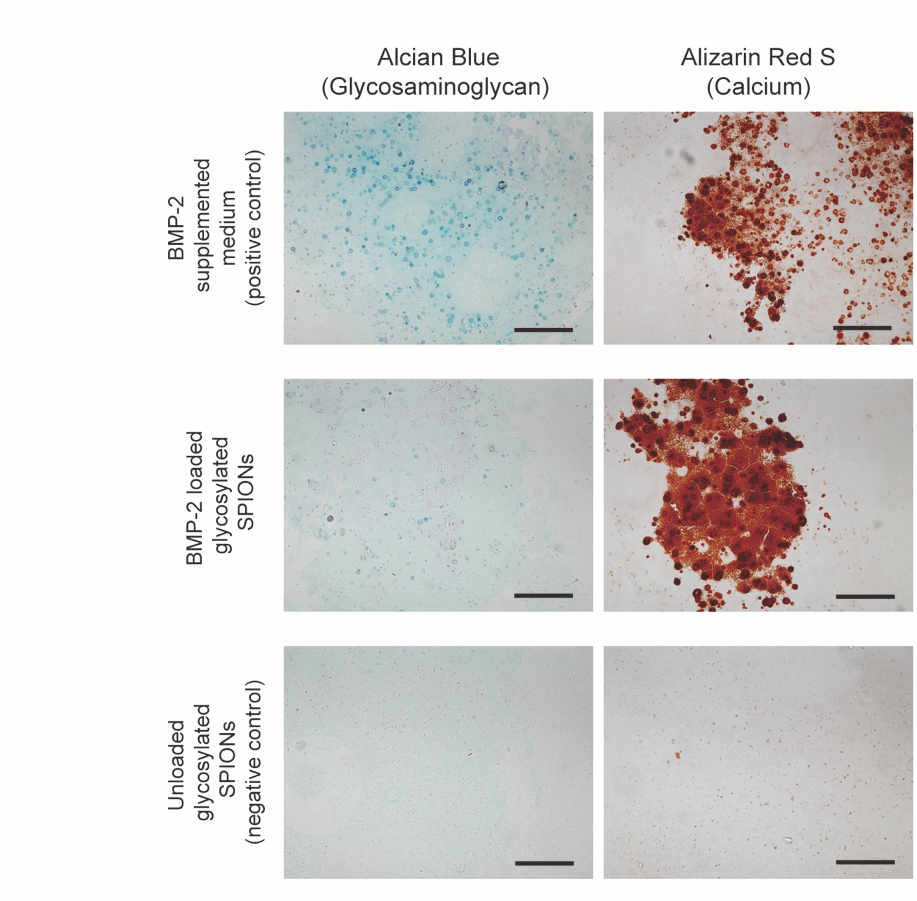


**Supplementary Figure 7.**

**Effect of BMP-2 delivery mode upon tissue mineralization. Tissue constructs were engineered for 28 days using hMSC-laden agarose hydrogels. Alizarin Red S staining showed deposition of calcium mineral (red) when BMP-2 was delivered in the medium or from glycosylated SPIONs, but not when exposed to unloaded SPIONs. Alcian Blue staining showed glycosaminoglycan (blue) in all cases. Scale bars = 500 µm.**

**
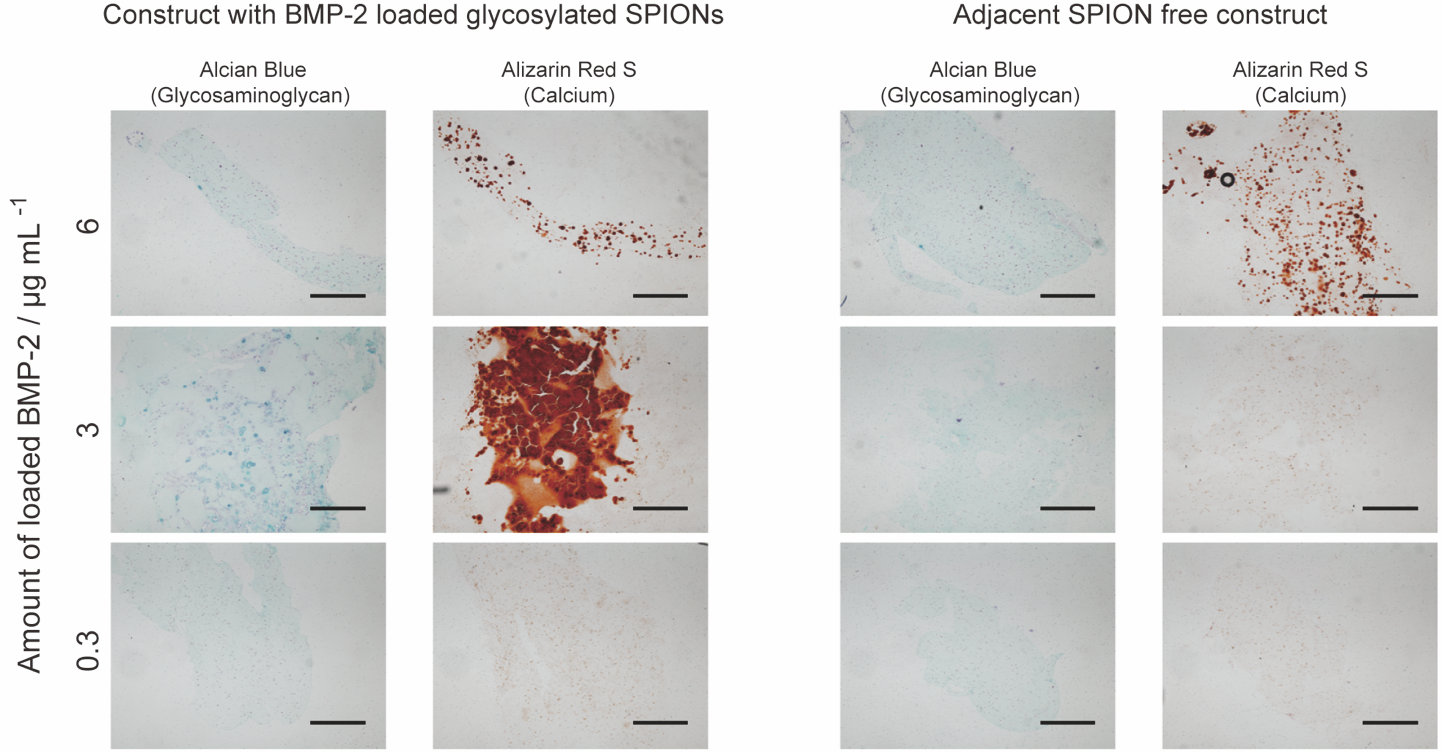
**

**Supplementary Figure 8.**

**Optimizing BMP-2 loading to confine tissue mineralization. Tissue constructs were engineered for 28 days using hMSC-laden agarose hydrogels with BMP-2 loaded glycosylated SPIONs, alongside adjacent hMSC-laden agarose hydrogels without any loaded SPIONs. Alizarin Red S staining showed deposition of calcium mineral (red) in both the SPION laden and the adjacent construct at the highest BMP-2 loading (6 μg per mL of hydrogel), only in the SPION laden construct at the intermediate BMP-2 loading (3 μg per mL of hydrogel) and in neither of the constructs at the low BMP-2 loading (0.3 μg per mL of hydrogel). Alcian Blue staining showed sulfated glycosaminoglycan (blue) in all cases. Scale bars = 500 μm.**


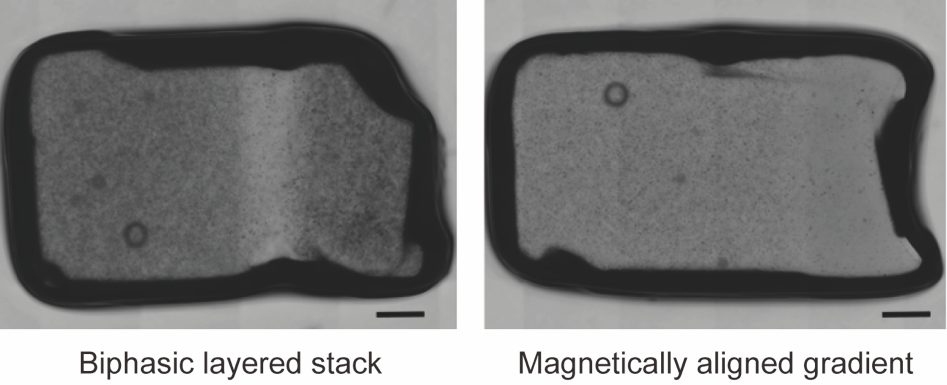


**Supplementary Figure 9.**

**Bright field microscopy images of a magnetically aligned gradient and a biphasic layered stack, both with hMSCs in agarose, captured immediately after gelation. The gradient material exhibits an even distribution of hMSCs throughout the construct, whereas a margin can be clearly observed between the two phases of a layered stack. Scale bars = 1 mm.**


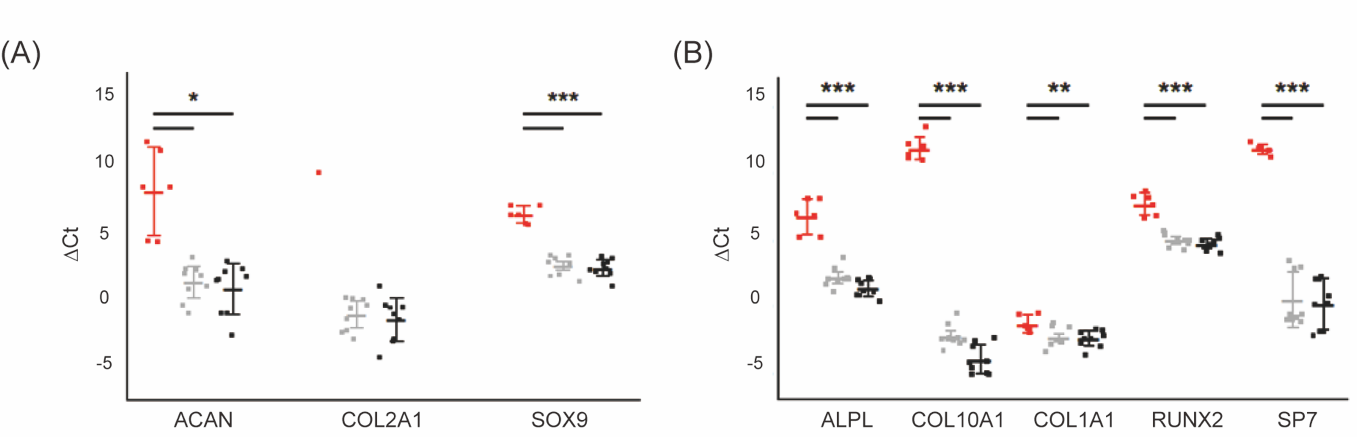


**Supplementary Figure 10.**

**Gene expression analysis of engineered osteochondral tissue. ΔCt values for the bone region (black), the cartilage region (gray) and a day 0 control (red) for genes associated with (A) cartilage formation and (B) bone formation (mean ± 95% confidence intervals, N = 3, n = 3 for 28 day constructs. N = 3, n = 1 for day 0 control; the *COL2A1* gene was only detected in one donor on day 0, but was detected for all 28 day samples). p < 0.05 (*), p < 0.01 (**), p < 0.001 (***).**

­­
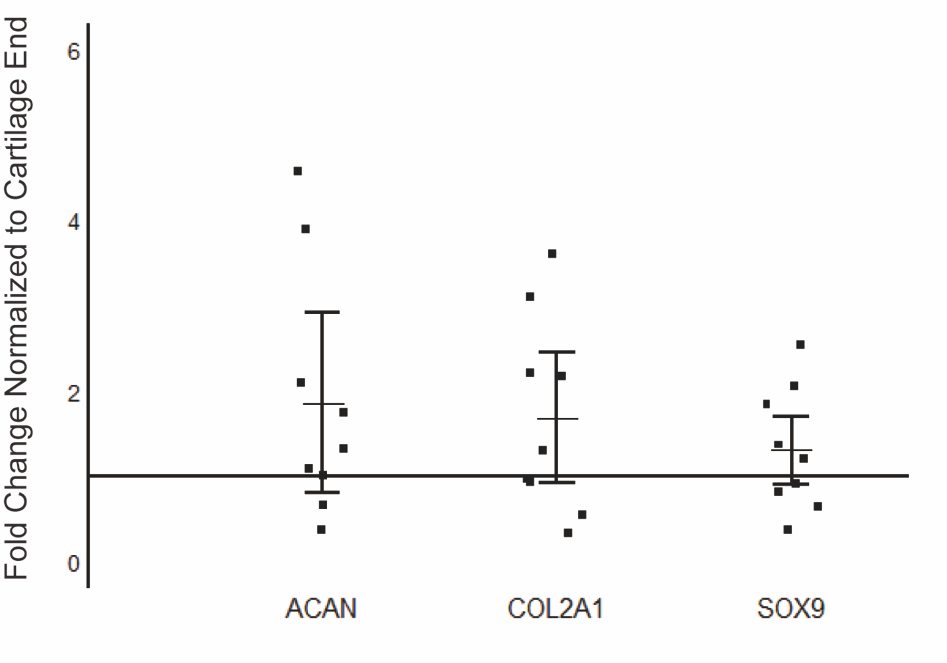


**Supplementary Figure 11.**

**Gene expression analysis of engineered osteochondral tissue. The bone region was normalized to the cartilage region for three cartilage-associated genes, showing no significant differences between the two regions. Comparison of differences were made using a single sample t-test (mean ± 95% confidence intervals, N = 3, n = 3).**

**
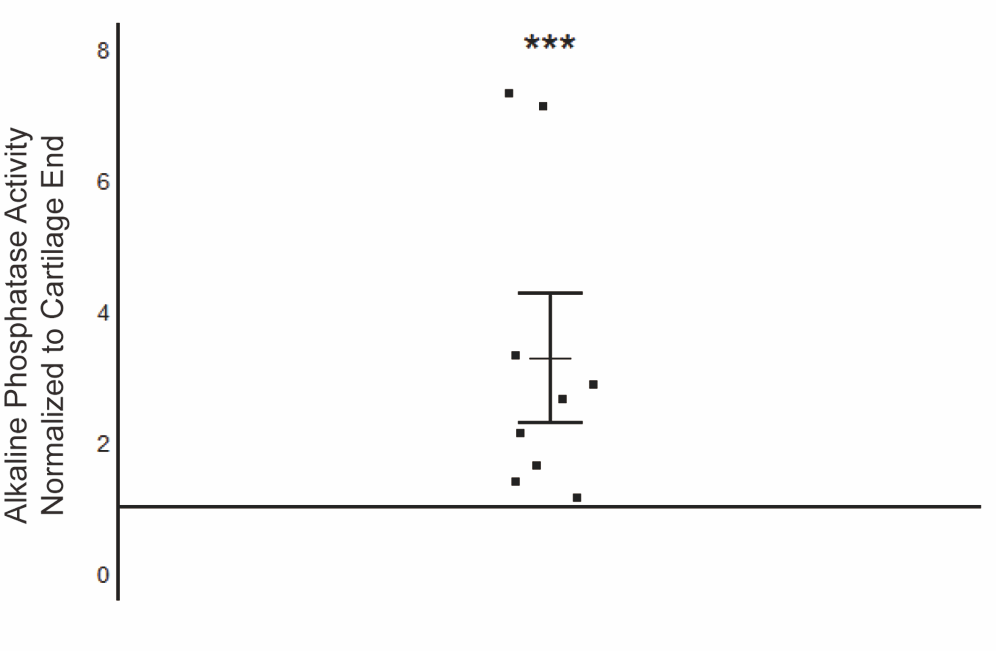
**

**Supplementary Figure 12.**

**Alkaline phosphatase measurements showing a significantly higher activity at the bone region, compared to the cartilage region. Comparison of differences were made using a one sample t-test after heteroscedasticity in the dataset was addressed (mean ±95% confidence intervals, N = 3, n = 3). p < 0.001 (***).**

**
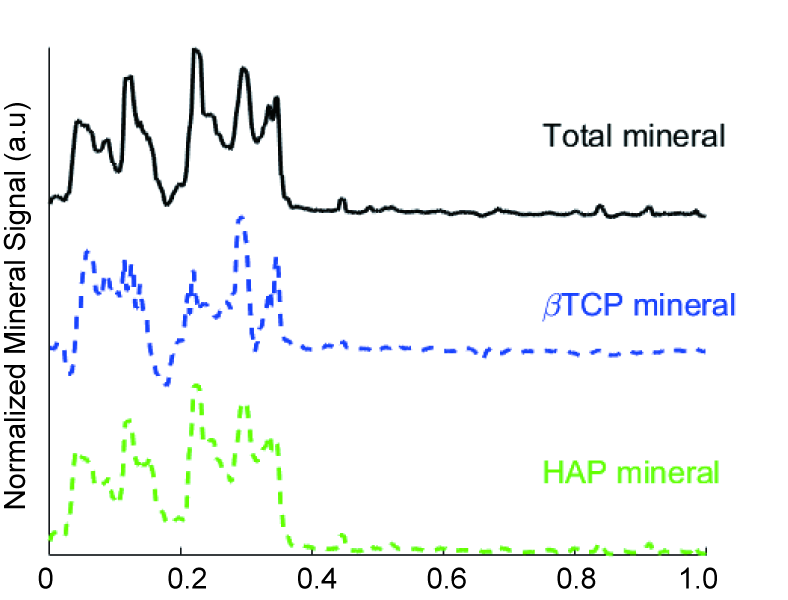
**

**Supplementary Figure 13.**

**Profile of Raman intensity corresponding to integrated signal from HAP and β-TCP (945 - 975 cm^-1^), HAP (ν_1_ PO_4_ at 962 cm^-1^) and β−TCP (ν_1_ HPO_4_^2-^ at 948 cm^-1^) across the length of the osteochondral tissue construct. The signals were offset to avoid overlapping.**

**
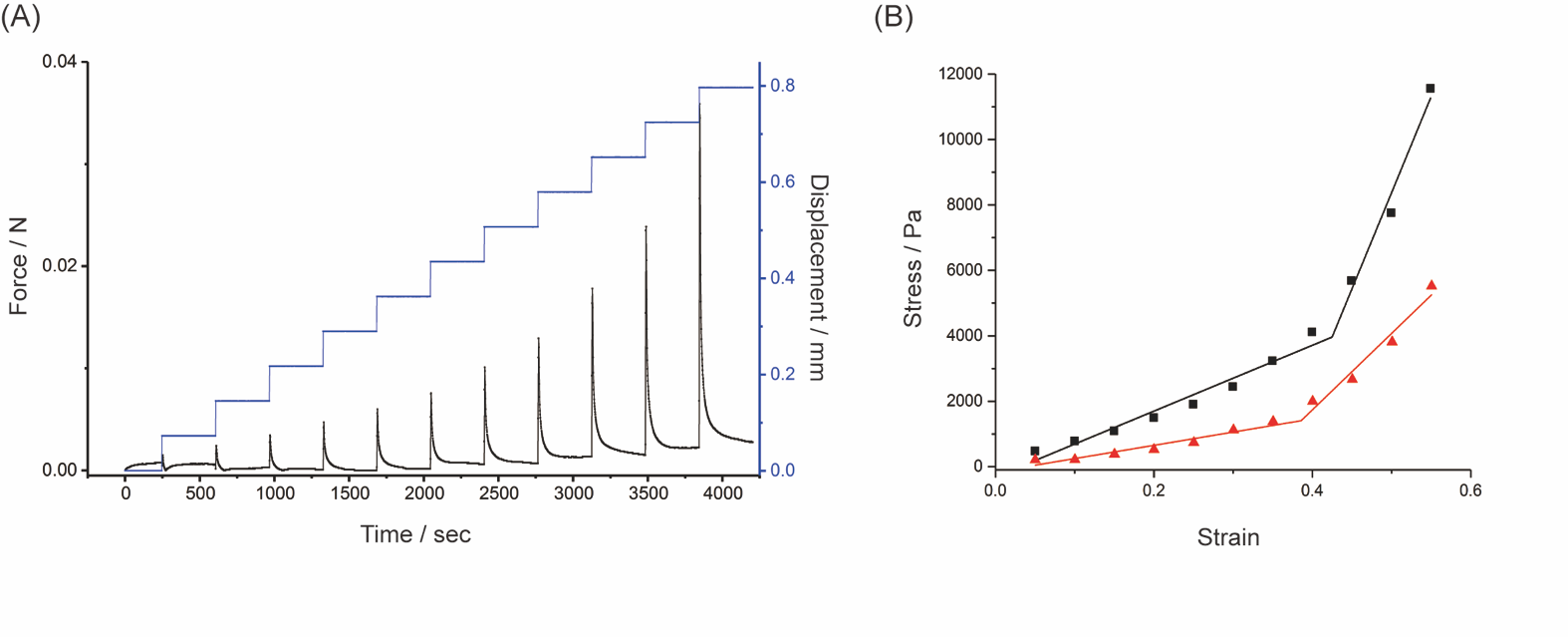
**

**Supplementary Figure 14.**

**Representative plots for the mechanical testing process. (A) Unconfined compression testing was performed using the engineered osteochondral tissue, with 12 steps of 5% strain increment applied. The exerted force (black trace, x axis) and displacement (blue trace, y axis) were measured. (B) The peak values of each compression cycle were used to plot an instantaneous stress-strain curve for the bone end (black square markers) and the cartilage end (red triangle markers), fitted to a bilinear model. The instantaneous modulus was calculated using the low-strain linear region only, to avoid any chance of artefacts from sample overload at high strain.**
